# Venous blood gas and biochemical analysis of wild captured green turtles (*Chelonia mydas*) and Kemp’s ridley turtles (*Lepidochelys kempii*) from the Gulf of Mexico

**DOI:** 10.1371/journal.pone.0237596

**Published:** 2020-08-12

**Authors:** Kerry L. McNally, Cody R. Mott, Jeffrey R. Guertin, Jonathan C. Gorham, Charles J. Innis

**Affiliations:** 1 Animal Health Department, New England Aquarium, Boston, Massachusetts, United States of America; 2 Inwater Research Group, Inc., Jensen Beach, Florida, United States of America; University of Minnesota, UNITED STATES

## Abstract

Blood was collected from wild captured green and Kemp’s ridley turtles off the west coast of Florida, USA. Blood gases and biochemical values were analyzed using a point of care (POC) device in the field. Analytes include pH, partial pressure of carbon dioxide (pCO_2_), partial pressure of oxygen (pO_2_), total carbon dioxide (TCO_2_), bicarbonate (HCO_3_), base excess (BE), oxygen saturation (sO_2_), lactate, sodium (Na), potassium (K), chloride (Cl), total carbon dioxide (TCO_2_), anion gap, ionized calcium, glucose, blood urea nitrogen (BUN), creatinine (Crea), hematocrit (Hct), and hemoglobin (Hb). These are novel data for wild healthy Kemp’s ridley turtles, and results for green turtles were generally consistent with past studies of green turtles with exceptions primarily in blood gas values. Ninety percent of the green turtles had fibropapillomatosis (FP), but none of the blood analytes were correlated with disease severity. Only BUN was correlated with weight of green turtles, and there was no correlation between blood parameters and weight of Kemp’s ridley turtles. This study provides data that are useful in understanding the physiologic status of sea turtles specific to this region, allowing for comparisons to other populations, life stages, and disease states.

## Introduction

The International Union for the Conservation of Nature (IUCN) lists the green turtle (*Chelonia mydas*) as Endangered [1] and the Kemp’s ridley turtle (*Lepidochelys kempii*) as Critically Endangered [2]. Threats to these species include habitat degradation, fishery interactions, pollution and illegal harvesting [1,2]. These sea turtle species are both found in the Gulf of Mexico, with the shallow seagrass beds along the northwest coast of Florida, U.S. being critical developmental habitat [3,4]. Juvenile green turtles in this area transition from being omnivores to herbivores, while Kemp’s ridley turtles are carnivores, primarily feeding on crustaceans [5].

Studying blood parameters of wild, presumed healthy animals is important for establishing baseline values. Such data are useful in assessing responses to stressors and disease conditions, and in the medical management and conservation of species. Several studies have evaluated blood gas, hematologic, and plasma biochemical values of green turtles from various regions of the world [6–16], but the Gulf of Mexico population has not been assessed. Blood data for healthy, wild Kemp’s ridley turtles are lacking as most studies have focused on captive, stranded or rehabilitated turtles [12,17–20], or turtles involved in fisheries interactions [21–23].

Portable point of care (POC) blood analyzers are useful for studying the physiologic status of wild animals, including sea turtles [24]. These devices can be used in the field, providing immediate results, and eliminating concern for transporting and/or storing samples for analysis [24,25]. The VetScan i-STAT^®^1 handheld analyzer (Abaxis Inc., Union City, CA) has proven to be valuable for field assessment of sea turtles, and for health assessment of turtles in captivity. It has been utilized for field studies of loggerhead turtles [26], leatherback turtles [27], hawksbill turtles [28], and green turtles [12,13], and for clinical studies of rehabilitating green, loggerhead, and Kemp’s ridley turtles [12,20,29].

The purpose of this study was to evaluate blood gas and biochemical parameters from green and Kemp’s ridley turtles in the Gulf of Mexico using the i-STAT analyzer in the field. In addition, this study evaluated correlations between various health parameters, morphometrics, and blood analytes.

## Materials and methods

### Ethics statement

This work was done under National Marine Fisheries Service Permit # 16598–03, Florida Fish and Wildlife Conservation Commission Permit # MTP-17-125A, and approved by the New England Aquarium Animal Care and Use Committee Protocol # 2017–07.

### Sample collection and processing

Green and Kemp’s ridley turtles were hand or dip-net captured from St. Martin’s Keys region off the coast of Crystal River, Florida, USA (28°50'24"N 82°45'00"W) from June 12 to June 17, 2017. As soon as a turtle was aboard the boat, body temperature of the turtle was taken using a digital laser infrared thermometer (Etekcity Corporation, Anaheim, CA) directed towards the pectoral region, and blood was collected from the dorsal cervical sinus through standardized protocols. Surface body temperatures were used instead of internal body temperatures (via cloacal temperature probe) to allow for temperatures to be taken immediately for use in blood analysis. Avoidance of cloacal temperature measurement also prevented contamination of the cloaca for subsequent microbiological sampling. Two captured turtles had cloacal temperatures performed for comparison, with similar temperatures recorded by both devices, allowing confidence in continuing with the infrared thermometer for the remainder of the study. The venipuncture site was disinfected with iodine and alcohol, and approximately 3 mL of blood was collected using 20 or 22 gauge needles and lithium heparin Vacutainer^®^ tubes (Becton, Dickinson and Company, Franklin Lakes, New Jersey, USA) for Kemp’s ridley turtles. Red top Vacutainer^®^ tubes were used for blood collection from green turtles, with two drops immediately transferred to a separate buffer for genetics, and the remainder of blood transferred to a lithium heparin Vacutainer^®^ tube. Using a 20 gauge needle on a 1 cc syringe, a small portion of blood was immediately retrieved from the lithium heparin tube for analysis on the VetScan i-STAT^®^1 handheld analyzer using the i-STAT CG4+ cartridge and CHEM8+ cartridge (Abbott Point of Care Inc., Abbott Park, IL) following manufacturers’ instructions. The analytes tested with the CG4+ cartridge include pH, partial pressure of carbon dioxide (pCO_2_), partial pressure of oxygen (pO_2_), total carbon dioxide (TCO_2_), bicarbonate (HCO_3_), base excess (BE), oxygen saturation (sO_2_), and lactate. The analytes tested with the Chem8+ cartridge include sodium (Na), potassium (K), chloride (Cl), total carbon dioxide (TCO_2_), anion gap, ionized calcium, glucose, blood urea nitrogen (BUN), creatinine (Crea), hematocrit (Hct), and hemoglobin (Hb).

The turtle’s body temperature was input into the analyzer to allow for calculation of temperature-corrected pH, pO_2_, and pCO_2_ given that the i-STAT analyzes blood at 37°C. Since the validity of the i-STAT temperature corrections has been questioned in other studies [26,30], corrections were also done manually based on equations suggested in previous publications [13,18,26,30,31] and shown below, where ΔT = 37°C–Turtle’s body temperature. ‘M’ subscript indicates manual correction and ‘I’ subscript denotes the instant values provided by the i-STAT.

$$pH_{M}=0.015(\Delta T)+pH_{I}$$

$$\mathrm{pC}O_{2M}=\mathrm{pC}O_{2I}(10^{‐0.019\mathrm{\Delta T}})$$

$$pO_{2M}=pO_{2I}(10^{‐0.0058\mathrm{\Delta T}})$$

Because pH has an effect on iCa calculation [13,18], the following formula was used to manually correct the iCa value for comparison to the i-STAT output [32]:

$$\mathrm{iC}a_{M}=\mathrm{iC}a_{I}(1+0.53(pH_{I}‐pH_{M}))$$

Following blood collection, captured turtles received physical examinations including morphometrics. The measurements obtained included straight minimum carapace length (SMCL), straight standard carapace length (SSCL), curved standard carapace length (CSCL), curved maximum carapace width (CMCW), straight maximum carapace width (SMCW), and straight midline plastron length (SMPL). Heart rates (beats per minute [bpm]) were taken using a Doppler blood flow detector (Pocket-Dop 3; Nicolet Vascular, Madison, WI). All turtles had Inconel 681 identification tags applied to the trailing edge of each front flipper and an internal passive integrated transponder (PIT) tag inserted into the right front flipper. All turtles were assessed for presence of fibropapillomatosis (FP) and assigned a total tumor score and Balazs tumor score based on the tumor measurements and quantity [33]. Once all exams were complete and samples collected, a number was painted on the carapace with a Paintstik® livestock marker to allow easy identification to avoid repeated sampling during the day. Turtles were typically released within 30–40 minutes (range 15–81 minutes on the boat) in the same area of capture.

### Statistical analysis

Statistical analysis was performed in R statistical software version 3.4.4 [34]. For each species, linear regressions were examined to identify relationships between the turtles’ weight, length, body temperature, or tumor score and the measured blood parameters. Influence on time of blood collection to cartridge insertion with blood parameters was also analyzed using linear regression. A Student’s t-test for normally distributed data was used to compare i-STAT auto-corrected data with manually corrected values. Only the iCa values were not normally distributed; thus, a Wilcoxon test was used to compare the i-STAT autocorrected data with manually corrected values for iCa. A p-value of < 0.05 was considered statistically significant following application of Bonferroni correction.

## Results

Twenty green turtles were sampled and had a mean time onboard the boat of 41 minutes (range 15 to 81 min). Five were captured by hand and the remaining 15 were captured with a dip net. Mean SSCL was 38.0 cm (range 31.0 to 47.0 cm) and mean weight was 7.0 kg (range 4.1 to 13.1 kg). Only two green turtles had no external evidence of FP, with the remaining green turtles having visible tumors (total tumor score mean 17.2 [range 0 to 38], Balazs score mean 1.6 [range 0 to 3]). Table 1 displays additional morphometric data.

**Table 1:** Mean, Standard Deviation (SD), median, and range (Min–Max) for health assessment information and morphometric data of sampled sea turtles separated by species.

	Kemp's ridley (n = 30)					Green (n = 20)				
Variable	Mean	SD	Median	Min	Max	Mean	SD	Median	Min	Max
**Time to blood (min)**	4	2	4	2	10	5	4	4	1	20
**Time to release (min)**	31	16	23	17	74	41	19	37	15	81
**Water Temp (°C)**	28.5	1.4	28.8	26.6	30.8	28.6	1.2	28.1	26.7	30.8
**Body Temp (°C)**	28.6	1.0	28.6	26.9	30.8	28.8	1.4	28.5	26.9	31.8
**HR (bpm)**	49	8	48	32	66	56	7	58	44	66
**Weight (kg)**	15.0	4.8	15.4	2.2	23.5	7.0	2.2	6.7	4.1	13.1
**Head width (cm)**	9.9	1.3	10.1	5.3	11.8	5.8	0.4	5.8	5.0	6.9
**SMCL (cm)**	45.2	6.1	46.6	24.4	52.7	37.1	3.8	37.5	30.4	46.0
**SSCL (cm)**	45.9	6.2	47.6	24.6	53.6	37.8	3.9	38.3	30.9	46.9
**SMCW (cm)**	44.4	6.7	45.1	22.0	52.9	30.0	3.1	29.9	25.6	38.9
**CSCL (cm)**	48.3	6.5	49.7	26.5	56.0	40.0	4.1	40.4	32.6	49.4
**CMCW (cm)**	49.4	7.1	51.1	25.8	58.6	34.3	3.5	34.1	29.1	42.7
**SMPL (cm)**	36.1	4.6	37.2	20.0	41.2	30.9	3.3	30.9	25.2	38.6
**Tumor score**	0	0	0	0	0	17.2	14.0	17.5	0.0	38.0
**Balazs Score**	0	0	0	0	0	1.6	0.8	2.0	0.0	3.0

SMCL = Straight minimum carapace length. SSCL = Straight standard carapace length. SMCW = Straight maximum carapace width. CSCL = Curved standard carapace length. CMCW = Curved maximum carapace width. SMPL = Straight midline plastron length.

Thirty Kemp’s ridley turtles were sampled and had a mean time onboard of 31 minutes (range 17 to 74 min). One Kemp’s ridley turtle had a SSCL of 24.6 cm and weight of 2.2 kg. The other 29 Kemp’s ridley turtles were more similar in size with a mean SSCL of 46.7 cm (range 34.3 to 53.6 cm) and mean weight was 15.4 kg (range 6.6 to 23.5 kg). All specimens were captured by hand except for the one smallest animal, which was captured with the dip net. All Kemp’s ridley turtles appeared clinically healthy with no visible injuries. Table 1 displays additional morphometric data.

Heart rates (HR) were successfully obtained from 27 Kemp’s ridley turtles and 20 green turtles (Table 1). The heart rates of green turtles had a mean of 56 bpm (range 44 to 66 bpm) and those of the Kemp’s ridley turtles had a mean of 49 bpm (range 32 to 66). There was no correlation of HR with time onboard the boat, capture method, temperature, body size within species, or any blood parameters (p > 0.05).

Time from capture to blood collection was a mean of four minutes for Kemp’s ridley turtles and five minutes for green turtles (Table 1). All Kemp’s ridley turtles had blood successfully collected, but collection was not successful for three green turtles. The CG4+ cartridge was run for 29 Kemp’s ridley turtles and 17 green turtles after a mean of 18 and 23 minutes since blood collection, respectively. The Chem8+ cartridge was run on 30 Kemp’s ridley and 17 green turtles after a mean of 16 and 21 minutes since blood collection, respectively. There was no significant correlation between the blood results with number of minutes from the time of blood collection to the time the cartridge was inserted into the device.

Table 2 and Table 3 display the analyte values from green and Kemp’s ridley turtles. Only BUN was positively correlated with weight in the green turtles (r^2^ = 0.467, p = 0.047). There were no correlations between blood parameters and size of Kemp’s ridley turtles (p > 0.05). No blood values were significantly correlated with temperature or tumor score (p > 0.05).

**Table 2:** Mean, Standard Deviation (SD), median, and range (Min–Max) of blood gas and lactate data provided by CG4+ cartridge for each species.

	Kemp's ridley (n = 29)					Green (n = 17)				
Variable (CG4+)	Mean	SD	Median	Min	Max	Mean	SD	Median	Min	Max
**i-STAT Temp (°C)**	28.4	0.9	28.6	26.7	30.0	28.4	0.9	28.5	26.9	30.0
**Blood to CG4+ (min)**	18	16	10	4	55	23	18	20	4	59
**pH**_**I**_	7.245	0.085	7.2	7.097	7.465	7.118	0.122	7.1	6.808	7.295
**pH**_**TC**_	7.362	0.090	7.358	7.204	7.585	7.228	0.129	7.210	6.901	7.419
**pH**_**M**_	7.373	0.085	7.368	7.222	7.583	7.246	0.123	7.228	6.937	7.428
**pCO**_**2I**_ **(mmHg)**	65.3	10.9	64.6	43.0	92.2	86.5	12.8	87.3	65.0	113.9
**PCO**_**2TC**_ **(mmHg)**	44.8	7.0	44.2	30.5	63.7	59.5	9.3	60.1	43.1	77.5
**pCO**_**2M**_ **(mmHg)**	44.9	7.0	44.1	30.5	63.7	59.5	9.3	60.1	43.1	77.5
**pO**_**2I**_ **(mmHg)**	78.8	11.2	80.0	43.0	98.0	66.5	10.9	67.0	49.0	90.0
**PO**_**2TC**_ **(mmHg)**	45.0	7.3	45.0	25.0	56.0	37.2	7.6	36.0	25.0	55.0
**pO**_**2M**_ **(mmHg)**	70.2	9.9	70.6	38.7	86.6	59.3	9.9	59.5	43.3	81.2
**BE (mmol/L)**	1	6	1	-8	16	0	6	0	-8	10
**HCO_3_ (mmol/L)**	28.3	4.6	28.6	20.0	39.9	29.3	4.5	29.2	22.2	36.9
**TCO_2_ (mmol/L)**	30	5	31	22	42	32	5	32	24	39
**sO_2_ (%)**	92	3	93	81	96	83	7	84	73	92
**Lac (mmol/L)**	10.48	3.60	10.87	3.77	17.96	17.82	3.70	19.97	11.12	21.00

Subscript I indicates values obtained instantly from the i-STAT. Subscript TC indicates temperature corrected values provided by the i-STAT device. Subscript M indicates manually corrected temperature values based on standard equations (see materials and methods). pCO_2_ = partial pressure of carbon dioxide, pO_2_ = partial pressure of oxygen, TCO_2_ = total carbon dioxide, BE = Base excess, sO_2_ = oxygen saturation, Lac = Lactate.

**Table 3:** Mean, Standard Deviation (SD), median and range (Min–Max) of chemistry and electrolyte values provided by Chem8+ cartridge for each species.

	Kemp's ridley (n = 30)					Green (n = 17)				
Variable (Chem8+)	Mean	SD	Median	Min	Max	Mean	SD	Median	Min	Max
**Blood to Chem8+ (min)**	16	14	10	4	51	21	17	15	4	55
**Na (mmol/L)**	157	3	157	150	163	157	4	158	149	165
**K (mmol/L)**	4.6	0.3	4.7	3.9	5.2	5.1	0.5	5.0	4.4	6.5
**Cl (mmol/L)**	117	4	118	107	128	115	4	115	106	122
**iCa (mmol/L)**	1.05	0.17	1.04	0.52	1.53	1.14	0.11	1.14	0.94	1.40
**iCa**_**M**_ **(mmol/L)**	0.96	0.16	0.95	0.48	1.40	1.05	0.10	1.05	0.86	1.29
**TCO2 (mmol/L)**	28	4	28	20	39	30	4	31	23	37
**Glu (mg/dL)**	75	9	74	63	99	77	10	77	61	97
**Glu (mmol/L)**	4.2	0.5	4.1	3.5	5.5	4.3	0.5	4.3	3.4	5.4
**BUN (mg/dL)**	67	13	66	44	100	3	2	2	2	8
**BUN (mmol/L)**	24.0	4.8	23.6	15.7	35.7	0.9	0.6	0.7	0.7	2.9
**Crea (mg/dL)**	0.1	0.0	0.1	0.1	0.2	0.2	0.1	0.2	0.1	0.3
**Crea (mmol/L)**	10.3	3.4	8.8	8.8	17.7	16.6	8.2	17.7	8.8	26.5
**Hct (%PCV)**	25	6	24	15	39	29	4	29	23	38
**Hb (g/dL)**	8.7	2.1	8.2	5.1	13.3	9.9	1.2	9.9	7.8	12.9
**AnGap (mmol/L)**	17	4	17	9	27	19	4	18	13	25

Subscript M indicates manually corrected temperature values based on standard equations (see materials and methods). Na = sodium, K = potassium, Cl = chloride, iCa = ionized calcium, TCO_2_ = total carbon dioxide, Glu = glucose, BUN = blood urea nitrogen, Crea = creatinine, Hct = hematocrit, Hb = hemoglobin, AnGap = anion gap.

The temperature corrected values for pH, pCO_2_, and the pH corrected value for iCa from the i-STAT analyzer were not significantly different from the manually corrected values (p > 0.05). The manually corrected pO_2_ was significantly higher than the i-STAT temperature corrected value (p < 0.001).

## Discussion

Studies that evaluate blood parameters from animals residing in different regions and habitats are valuable because they provide insight on the breadth of values that might be expected within a given species. This study is the first to evaluate these specific blood parameters for wild green turtles from this region and healthy, wild Kemp’s ridley turtles. Compared to previous studies [6,8,13,16,22,23], results were generally within expected ranges for sea turtles with some exceptions described below. The maximum sodium values in this study (e.g. 163–165 mmol/L) may be considered slightly high in some contexts, but likely illustrate natural variation.

For green turtles, there was some variation in blood values from this study compared to other studies. Blood gas and pH results in this study were somewhat different compared to green turtles in the Galapagos, including a lower pH and pO_2_, and a higher pCO_2_ in the present study [13]. Potassium, iCa, and lactate were all higher in green turtles in this study compared to Galapagos green turtles [13], but potassium was comparable to values reported in other studies [6,22]. Glucose values vary widely among studies, with green turtles in the present study having a mean in the middle of previously reported ranges [6,11,13,16,22]. Differences between the Galapagos study and the present study may be due to a variety of factors. For example, different body temperatures, activity levels, dive durations, metabolic rates (as related to body size, temperature, and feeding activity), and exertion during capture could easily produce differences in pH, blood gases, lactate, and potassium between studies [17,18,22,26,27,35]. In turtles, the blood pH is expected to be lower, and blood gas values are expected to be higher at higher body temperatures [18,36]. In this study, mean body temperature was 8°C higher than in the Galapagos study. The relatively high lactate concentrations in the present study may also indicate some degree of anaerobic metabolism and metabolic acidosis as related to pre-capture behavior (e.g., if the turtle had been on a prolonged dive) or due to exertion during capture and processing.

BUN was similar [8] or lower [16,22] in green turtles in this study compared to some previous studies of free-ranging green turtles. BUN is a dynamic analyte in sea turtles, showing wide variation based on diet, disease conditions, and reproductive status, although the biochemical mechanisms for these variations are not fully understood [6,7,16,18,19,27]. A higher BUN is typical in animals fed a high-protein diet [16,37], which may account for the large difference in BUN between carnivorous Kemp’s ridley turtles and herbivorous green turtles observed here. The Kemp’s ridley turtles captured were immature and had recruited to neritic habitats, which typically occurs between 20 to 25 cm SSCL [3]. Kemp’s ridley turtles at this life stage have a diet primarily consisting of crabs and other crustaceans [5]. The juvenile green turtles captured consisted of a size range indicating a complete shift to herbivory, with a diet primarily consisting of seagrasses and algae, although some animal matter such as jellyfish may also be consumed [5]. The relatively low BUN concentrations in green turtles may reflect a low protein diet confirming the shift to herbivory [16]. BUN values for apparently healthy green turtles vary as much as ten-fold among various studies, and controlled studies in captivity indicate that diet has a major effect [16]. Since many studies, including the present study, did not determine the diet or reproductive status of the animals, it is impossible to determine the reason for BUN variability. However, it seems likely that local food items, proportion of dietary animal matter vs. plant matter, migratory status, season, health status, and reproductive status were not uniform across these studies, likely influencing the BUN concentrations.

There are limited blood parameter studies of wild healthy Kemp’s ridley turtles. Most studies of this species focus on stranded or rehabilitated turtles [18–20,38]. The closest studies on wild healthy Kemp’s ridley turtles have been opportunistic due to incidental captures from fishery interactions [22,23]. In this study, glucose and lactate values of Kemp’s ridley turtles were lower than other studies [22,23]. This could be due to the greater physiologic stress of fishery interactions compared to the capture method in this study [39]. Other parameters that were tested in each of these studies, including BUN, potassium, sodium, and chloride, were similar [22,23]. Blood gases of Kemp’s ridley turtles have only been reported in captive, stranded, or rehabilitated turtles [17,18,20,29]. The pH values were lower in this study and were more similar to stranded animals than convalescent [18,20]. As seen in leatherback sea turtles, these slightly acidotic values, which were also seen in the green turtles, may be a response to capture or prolonged submergence pre-capture [27,35], but may also be normal for this temperature range and location.

Blood parameters have been correlated with carapace length and weight in sea turtles to indicate health status, life stage or reproductive status [11,15,40]. There was no correlation of turtle carapace length with blood parameters for either species. In Galapagos green turtles and stranded loggerhead sea turtles, lactate was seen to be positively correlated with carapace length [13,41]. The lack of correlation in this study could be due to the small range and similar age class within the species groups. BUN was positively correlated with weight of the green turtles, but not Kemp’s ridley turtles. This correlation may be skewed in this study due to the low sample size, with the two largest green turtles with a higher BUN being outliers, but the numerous variables that affect BUN, as previously described, may play a role in the slight significance. Other studies that compared blood data with the size of the turtles did not evaluate BUN [11,15], so further understanding of this correlation would be useful through future studies with increased sample sizes. Several parameters not tested in this study, such as albumin, total protein, uric acid, and various hematological values, have also been correlated to body size (carapace length and/or weight) in other studies [6,9,10,15].

There was a high prevalence of FP observed in the captured green turtles of this study, with 90% of the green turtles having some degree of external tumors. There was no correlation between any of the blood parameters evaluated in this study with total tumor score or Balazs tumor score. Other studies have reported increased BUN concentrations with tumor severity, which was not seen in this population [7]. Some of this discrepancy may be due to the low sample size of green turtles in this study. Blood parameters not tested here that have been observed to correlate to FP severity include total protein, total globulins, calcium, and phosphorous [4,7].

The i-STAT device proved adequate for this field study. There were a low number of complications, with only 4 cartridge errors due to insufficient sample insertion, temperature out of range, or positioning errors. Maintaining the environmental conditions necessary for proper functions of the i-STAT analyzer, such as temperature and humidity, were a challenge that was overcome by keeping the equipment in a shaded area and near an ice pack for temperature control. Although pH, pCO_2_, and iCa values were manually corrected for temperature using previously published calculations, the auto-corrected values from the i-STAT were very similar and clinically acceptable as seen in other studies [13,28]. Hematocrit determined by the i-STAT analyzer may be lower than that determined by conventional centrifugation, so these values should be reviewed cautiously [24,28,42].

Venous pO_2_ data are important in the evaluation of critically ill sea turtles [43,44]. Although we did not maintain strict anaerobic conditions during sample processing, clinically useful pO_2_ data have been derived using a similar method in mammals [45–47]. However, the temporal delay in some analyses in this study may have resulted in artifactual changes in pO_2_, as recently described for another turtle species [48]. Blood analysis was conducted as soon as possible after the blood was collected (Table 2, Table 3) with the longest lag time of 59 minutes. Amount of time between blood collection and time the cartridge was inserted into the device had no significant impact on blood values. While there did not appear to be artifactual changes due to the varying time from blood collection to cartridge analysis, it is recommended to perform analysis as soon as possible after blood is drawn. In the present study, the manually corrected pO_2_ was significantly different from the auto-corrected i-STAT values. This discrepancy has been seen in other studies and is likely due to differences in hemoglobin-oxygen affinity between sea turtles and mammals. The i-STAT temperature correction formula for pO_2_ relies on mammalian hemoglobin-oxygen affinity, making it inaccurate for ectotherms [30]. Cumulatively, these considerations suggest that pO_2_ data from this study should be interpreted cautiously.

## Conclusion

This study provided blood gas and biochemical analysis in the field for two species of wild captured sea turtles. This was the first analysis of these blood parameters for wild healthy Kemp’s ridley turtles, and for green turtles in the Gulf of Mexico. This study is important for future comparisons and understanding the health status of these endangered species.

## Supporting information

S1 Table Raw data of sampled turtles including morphometrics and blood values. (XLSX)
